# Case Report: Cabezas syndrome caused by *CUL4B* gene mutations in two unrelated Chinese boys

**DOI:** 10.3389/fnins.2025.1600852

**Published:** 2025-07-21

**Authors:** Li Lin, Qi Yang, Shujie Zhang, Xunzhao Zhou, Xiaoling Li, Sheng Yi, Qiang Zhang, Shang Yi, Sheng He, Zailong Qin, Jingsi Luo

**Affiliations:** ^1^Guangxi Clinical Research Center for Birth Defects, Guangxi Key Laboratory of Reproductive Health and Birth Defects Prevention, Maternal and Child Health Hospital of Guangxi Zhuang Autonomous Region, Nanning, China; ^2^Department of Genetic and Metabolic Central Laboratory, Maternal and Child Health Hospital of Guangxi Zhuang Autonomous Region, Nanning, China; ^3^Department of Child Health Care, Maternal and Child Health Hospital of Guangxi Zhuang Autonomous Region, Nanning, China; ^4^Guangxi Clinical Research Center for Pediatric Diseases, Maternal and Child Health Hospital of Guangxi Zhuang Autonomous Region, Nanning, China

**Keywords:** *CUL4B*, Cabezas syndrome, intellectual disability, developmental delay, seizures, novel variant

## Abstract

As a component of the ubiquitin ligase complex, Cullin 4B (CUL4B) is involved in the process of ubiquitination of different substrates, controlling genome stability, nucleotide excision repair, and chromatin-remodeling. The mutations in the *CUL4B* gene are revealed to be a cause of Cabezas syndrome (OMIM 300354), a rare syndromic form of X-linked intellectual disability (XLID). In this study, whole-exome sequencing analysis and Sanger sequencing identified two maternally inherited likely pathogenetic variants (*CUL4B*, NM_001079872.2: c.803dupT/p. Leu268fs*5; c.953_957delTTATA/p. Ile318fs*2) in two probands, respectively. Patients carrying *CUL4B* variants presented with broad and variable phenotypic defects. The clinical manifestations of the two boys are consistent with Cabezas syndrome; however, they exhibit significant heterogeneity compared to previously reported cases. Phenotypic manifestations resulting from genetic variations may exhibit population differences and, in some cases, may present with concealed or latent expressions. Therefore, regular pediatric health check-ups and appropriate molecular diagnostic techniques are essential for the early detection, diagnosis, and treatment of such disorders. Our findings could be used to better define the genetic map in this area and will be valuable in the genetic diagnosis of the disease.

## Introduction

CUL4B, a member of the cullin-RING ubiquitin ligase (CRL) family, is the largest E3 ligase subtype in mammals. CUL4B, as a key scaffold protein for the assembly of cullin 4B-RING ubiquitin ligase (E3) complexes (CRL4B), assembles CRL4B using adaptors [Rbx1 and DNA damage-binding protein 1 (DDB1)] and DDB1-cullin-associated factors (DCAF) substrate receptors (Zheng et al., 2002). CRL4B regulates a wide range of cellular processes through the ubiquitination modification and proteasome degradation of substrates, such as cell cycle regulation, degradation of cellular proteins, signal nucleotide excision repair, embryonic development, and DNA damage response control (Liu et al., 2012; Londin et al., 2014). Loss of functions due to the *CUL4B* mutations might impact their assembly or catalytic activity of CRL4B-based E3 ligase complexes, leading to abnormal interactions between CRL4B and substrate proteins. The clinical phenotypes caused by these mutations vary widely due to their tissue- and cell-specific effects (Stier et al., 2023; Kerzendorfer et al., 2011). They are usually characterized by abnormal growth and development, including intellectual disability, short stature, and malformations. Cabezas syndrome (OMIM 300354), a rare syndromic form of X-linked intellectual disability (XLID), was revealed to be caused by *CUL4B* mutations (Cabezas et al., 2000). In addition to intellectual disability, typical manifestations of Cabezas syndrome include motor delay, short stature, a prominent lower lip, small testes, muscle wasting in the lower legs, and other variable features (Cabezas et al., 2000; Okamoto et al., 2017). To date, over 90 cases carrying *CUL4B* variants have been described in *CUL4B,* including missense, frameshift, splicing, and primary truncation variants (Londin et al., 2014; Cabezas et al., 2000; Okamoto et al., 2017; Isidor et al., 2009; Tarpey et al., 2007; Ravn et al., 2012; Badura-Stronka et al., 2010; Vulto-van Silfhout et al., 2015; Tzschach et al., 2015; Weissbach et al., 2017; Lopez et al., 2020; Della Vecchia et al., 2023; Magalhaes et al., 2023; Nakamura et al., 2019; Zou et al., 2007). Here, we report two frameshift variants in the *CUL4B* gene in Chinese families and explore the heterogeneity of phenotypes of *CUL4B* variant carriers through a review of previously reported cases.

## Materials and methods

### Patients

#### Clinical features

Two male pediatric patients from unrelated, non-consanguineous Chinese families were referred to genetic counseling to investigate unexplained seizures and severe global developmental delay.

Case 1, a 3.5-year-old boy, was born at full term to unrelated parents and had a history of treatment for neonatal pneumonia after birth. He was able to hold his head up steadily at 1 year old, could sit independently by age 2, and started walking alone at 2.5 years old. However, he still cannot feed himself. His language development was also notably delayed. He had not yet begun to speak. Seizures started at 3.5 years of age in sleep (sudden rolling of the eyes, increased muscle tone in the limbs, and cyanosis). The frequency of seizures was 2–3 times a day, and the duration ranged from 10 s to 4 min. After the remission of epilepsy, children also have involuntary limb convulsions. The 24-h electroencephalogram (EEG) revealed abnormal infantile electroencephalographic topography: Background activity was slowed, with slow-wave discharges more prominent in the posterior regions, particularly in the occipital area. Brain MRI shows widened extracerebral spaces bilaterally in the frontotemporal regions, deepened sulci in the frontal and top regions, and a slender appearance of the splenium of the corpus callosum. Physical examination revealed developmental delays [weight 12.4 kg (<−3SD), length 80.8 cm (<−3SD), and head circumference 48.5 cm] and mild dysmorphia features (low nasal bridge, low-set ears, micrognathia, and brachydactylia). Muscle tension showed hypotonia. Physiological reflexes were present. The patient underwent a Gesell Developmental Schedules (GDS) score test at the age of 3 years. The adaptive behavior quotient was 28 points (significant developmental delay), with a personal-social behavior quotient of 30 (significant developmental delay), and the gross motor quotient was 43 points (moderate developmental delay) with a fine motor quotient of 36 (significant developmental delay); language ability was worse than that of his peers, with a development quotient (DQ) of 26 points (significant developmental delay). In addition, this proband presented with recurrent respiratory infections after birth. The ECG displayed an incomplete right bundle branch block with T-wave changes.

Case 2 was a male patient who was first seen at our hospital at the age of 9 months, presenting with global developmental delay and cerebral dysgenesis. The patient was born at 39 weeks’ gestation with normal weight (3,600 g) and height (50 cm). He had a history of asphyxiation and resuscitation at birth, with Apgar scores of 7/9. The patient presented with a 4-month history of global developmental delay. He began raising his head at 4 months and rolling over at 6 months. At his visit at 9 months of age, the patient cannot sit independently or crawl, but can laugh when teased and can grasp objects voluntarily. At physical examination, he had a weight of 66.2 cm (<−2SD), a length of 7.5 cm (<−2SD), and a head circumference of 42.3 cm (<−2SD). The color ultrasonography revealed right-sided cryptorchidism. The 3-h video EEG showed an abnormal infantile electroencephalographic topography: bilateral posterior head slow waves and sharp slow wave discharges, particularly pronounced in the occipital region. No other abnormalities were found on the brain MRI, ECG, visual evoked potential (VEP), and auditory evoked potential (AEP). GDS scores show mild developmental delay in adaptive behavior (58), personal-social behavior (63), and gross motor (61), fine motor (60), and language abilities (58). The patient’s uncle is an individual with intellectual disability.

We collected physical examination and laboratory test results during their hospital visits. DNA samples were extracted from the peripheral blood of the two children and their parents to detect potential variants using index case whole-exome sequencing (WES) and Sanger sequencing. The project was approved by the ethics committee of the Maternal and Child Health Hospital of Guangxi Zhuang Autonomous Region. Informed consent was obtained from the parents/guardians of the children for whole-exome sequencing, Sanger sequencing, and publication of photographs on behalf of the proband.

#### Genetic analysis

The potential variants were considered following the alignment of the patient genome sequence against the ClinVar,^1^ HGMD,^2^ HPSD,^3^ ExAC,^4^ 1000G,^5^ and the SNP^6^ databases. PolyPhen 2.0 and Mutation Taster tools were used to evaluate the pathology of the potential missense variants. Sanger sequencing was employed to further validate the candidate genes and to identify the inheritance pattern of the variant. The pathogenicity of the variants was classified following the ACMG/AMP guidelines (Richards et al., 2015).

## Results

### Molecular analysis

**Figure 1:**
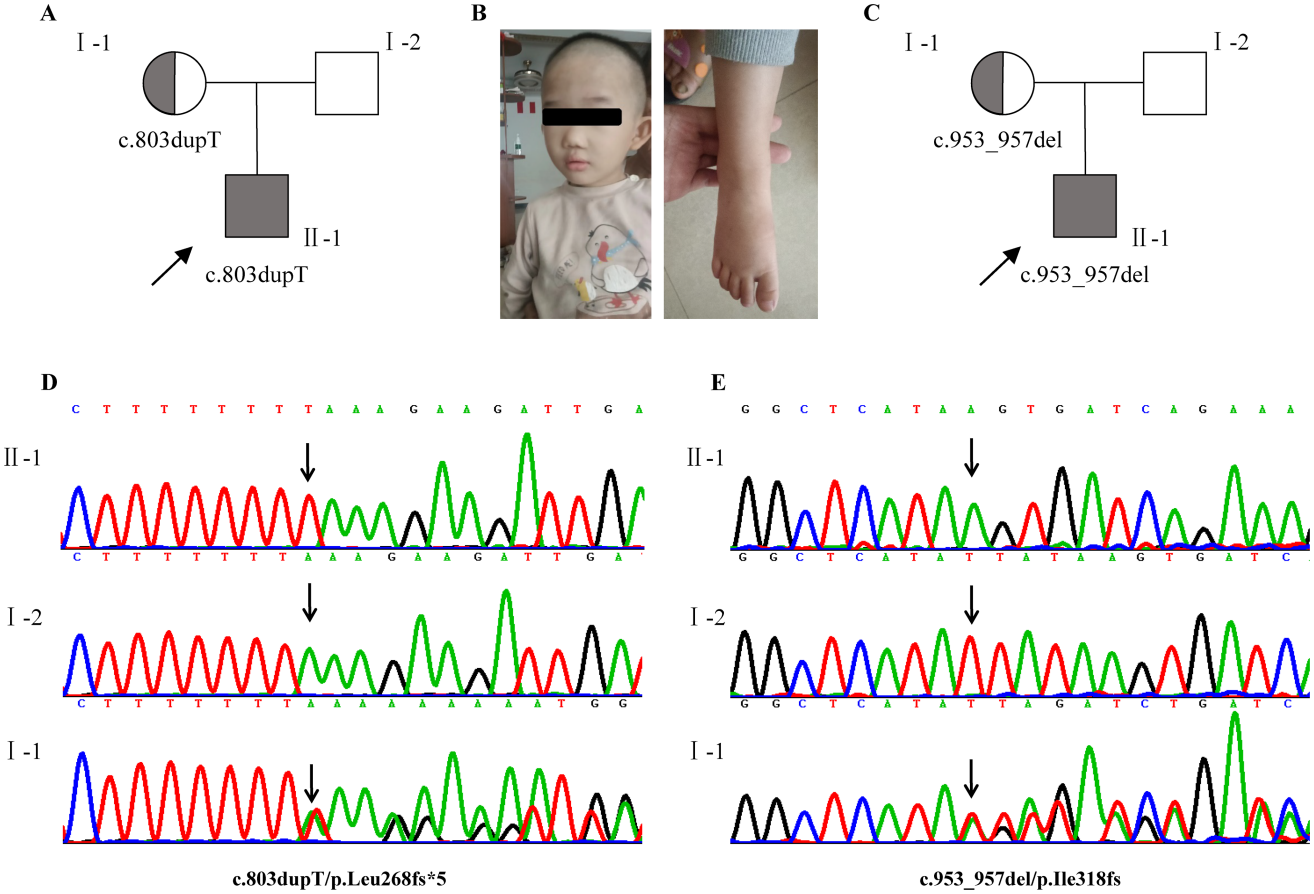
Clinical and genetic features. **(A,C)** Pedigrees of affected families 1 and 2. **(B)** Photograph of case 1 at the age of 3 years 6 months showing mild craniofacial dysmorphic features, brachydactyly. **(D,E)** Two heterozygous frameshift mutations (NM_001079872.2, c.803dupT/p. Leu268fs*5; c.953_957delTTATA/p. Ile318fs*2) in CUL4B were identified in cases 1 and 2, respectively. Sanger sequencing confirmed that both of them were inherited from the mother. Probands are denoted by arrows. Gray indicates that the individual is affected.

Two heterozygous frameshift mutations (c.803dupT/p. Leu268fs*5; c.953_957delTTATA/p. Ile318fs*2) in *CUL4B* (NM_001079872.2) were identified in cases 1 and 2, respectively. Among them, c.803dupT/p. Leu268fs*5 was a novel mutation. Sanger sequencing confirmed that the mothers of the two cases were both asymptomatic carriers of the same mutation. Mutation Taster predicts that both mutations are damaging. According to the ACMG/AMP guideline, the two variants are classified as likely pathogenic as the following supporting evidence PVS1 + PM2_, which is applied to it. These variants are absent from the general population (AF in gnomAD is 0); thus, PM2 is supported. *CUL4B* is a haploinsufficient gene (HI score = 3), and frameshift mutations in its coding region can lead to a loss of function, thereby causing Cabezas syndrome (Thus, PVS1 applied) (Zheng et al., 2002; Della Vecchia et al., 2023; Magalhaes et al., 2023; Nakamura et al., 2019) (Figure 1).

## Discussion and conclusion

Cabezas syndrome is a rare inherited X-linked neurodevelopmental disorder caused by *CUL4B* variants. To date, many individuals with Cabezas syndrome have been reported worldwide, but sporadic cases have been found in China. In this study, we reported two Chinese boys with global developmental delay and seizure caused by two maternal variants (c.803dupT/p. Leu268fs*5 and c.953_957delTTATA/p. Ile318fs*2) in the *CUL4B* gene. Both variants were first reported in the Chinese population. The variant (c.803dupT/p. Leu268fs*5) was first reported worldwide.

The novel variant (c.803dupT/p. Leu268fs*5) was located in the exon 4 of the *CUL4B* gene, which is the DNA damage-binding protein 1(DDB1)-binding domain (exon4-5) in the N terminus of *CUL4B* (Liu et al., 2012). DDB1 is a necessary linker protein for Rbx1 binding and subsequent recruitment of the E2 ubiquitin-conjugating enzyme. The patients for which the mutations were identified in exon 4–6 shared overlapping phenotypic features with the case 1 examined here, including short stature, abnormal muscle tone, severe developmental delay in intellectual and speech, and brachydactyly (Londin et al., 2014; Tarpey et al., 2007; Vulto-van Silfhout et al., 2015; Weissbach et al., 2017; Nakamura et al., 2019; Vitale et al., 2001). We believe that the change in the binding domain of CUL4B due to mutations may affect its connection to DDB1, resulting in failure to bind to substrate recognition proteins (Liu et al., 2012).

Previous studies indicate that 43% of *CUL4B* patients develop seizures unrelated to the types of mutation (Della Vecchia et al., 2023). In our study, Case 1 experienced recurrent seizures from the age of 3.5 years. In contrast to the types of seizures previously reported in patients, such as self-limited febrile seizures (Della Vecchia et al., 2023), this patient experienced a more unusual type of seizure in Cabezas syndrome, namely nocturnal focal tonic seizures. Although the seizure pattern of Case 1 is similar to that reported in Italian cases (Della Vecchia et al., 2023), no sub-continuous and widespread discharges in sleep were observed in the 24-h EEG. Slow-wave discharges in the occipital region were also observed in Case 2, although this patient did not have seizures. According to the published cases, the age at seizure onset is within 4–5 years of age; therefore, we cannot exclude the possibility of seizures of Csea2 (Della Vecchia et al., 2023). The potential mechanisms underlying *CUL4B* seizures could be linked to the presence of a malformation of cortical development, or may result from the detrimental effects of loss-of-function of the *CUL4B* gene at the cellular level (Vulto-van Silfhout et al., 2015; Della Vecchia et al., 2023). The neural precursor cells of nervous system-specific Cul4b knockout mice (Cul4b^Nestin-Cre^ mice) showed an increased tendency to differentiate into glial fibrillary acidic protein (GFAP)-positive cells, where GFAP is recognized as a marker of mature astrocytes. Astrocyte dysfunction is associated with various neurodevelopmental disorders (Zhao et al., 2015). In another CUL4B-deficient mouse model (Cul4b^Sox2-Cre^ mice), a reduction in parvalbumin (PV)-positive neuron numbers and altered dendritic morphology were observed. These changes suggest impaired inhibitory regulation and diminished dendritic integration capacity in hippocampal neural circuits, ultimately leading to increased epileptic susceptibility and compromised spatial learning ability (Chen et al., 2012). Furthermore, the interaction of CUL4B with the substrates related to brain malformations and neurodegeneration may play another role (Haouari et al., 2022). Neural precursor cell expressed developmentally down-regulated gene 4-like (NEDD4-2) encodes a ubiquitin E3 ligase involved in epileptogenesis. It revealed that Nedd4-2 haploinsufficiency caused increased susceptibility and severity of pentylenetetrazol (PTZ)-induced seizures in mice (Liu et al., 2021; Wu et al., 2015). Functional studies on the effect of pathogenic variants identified in patients with seizures would be needed to validate the hypotheses.

We reviewed the phenotypes of nearly 100 patients from 36 families that have been reported to date (Table 1). Patients with Cabezas syndrome exhibit overlapping phenotypes, including mental retardation (82/83, 98.8%), speech delay (72/74, 97.3%), motor delay (57/58, 98.3%), behavioral problems (42/60, 70%), short stature (56/70, 80.0%), hypogonadism (45/64, 70.3%), and toe deformity (53/68, 77.9%) (Londin et al., 2014; Cabezas et al., 2000; Okamoto et al., 2017; Isidor et al., 2009; Tarpey et al., 2007; Ravn et al., 2012; Badura-Stronka et al., 2010; Vulto-van Silfhout et al., 2015; Tzschach et al., 2015; Weissbach et al., 2017; Lopez et al., 2020; Della Vecchia et al., 2023; Magalhaes et al., 2023; Nakamura et al., 2019; Zou et al., 2007). Moreover, the clinical manifestations associated with *CUL4B* gene variants are broad and exhibit significant heterogeneity. Patients can also present with intention tremor, increased peripheral blood mononuclear cells (Zou et al., 2007), craniofacial abnormalities, gait abnormalities, white matter lesions, and cortical dysplasia, among others. This phenotypic heterogeneity appears to have little correlation with the type and location of the mutations (Londin et al., 2014; Cabezas et al., 2000; Okamoto et al., 2017; Tarpey et al., 2007; Vulto-van Silfhout et al., 2015; Weissbach et al., 2017; Lopez et al., 2020; Della Vecchia et al., 2023; Magalhaes et al., 2023; Nakamura et al., 2019; Zou et al., 2007; Zhao et al., 2015). It is interesting to note that phenotypic manifestations resulting from genetic variations may vary among different populations, and in some cases, may present with concealed or latent expressions (Okamoto et al., 2017; Weissbach et al., 2017; Nakamura et al., 2019). Data showed that 73.9% (17/23), 87.5% (35/40), 82.0% (32/39), 67.4% (29/43), and 67.2% (41/61) of European populations (3, 8-17) presented with prominent forehead, abnormal ears, narrow palpebral fissure, low nasal bridge, and prominent lower lip, respectively, while only 10–20% of Asians presented with craniofacial deformities (Okamoto et al., 2017; Nakamura et al., 2019; Zou et al., 2007). Macrocephaly, pes cavus, wasted lower-leg muscles, kyphosis, and strabismus have also not been reported in the Chinese population with Cabezas syndrome. Additionally, in case 2, the patient appeared to exhibit significant global developmental delay rather than other dysmorphisms when compared to previously reported cases in Europe (Tarpey et al., 2007; Vulto-van Silfhout et al., 2015) (Table 2). These findings revealed that the phenotypes of Cabezas syndrome in the Chinese population tend to present in an atypical form, suggesting that Cabezas syndrome might be clinically underdiagnosed. Furthermore, some scholars have suggested that the phenotypic expression in affected individuals might be age-dependent: among those under the age of 10 years, the rates of obesity, tremor, gynecomastia, and hypogonadism are significantly lower (Nakamura et al., 2019). The lack of some clinical features may pose a clinical diagnostic challenge. Therefore, regular pediatric health check-ups and comprehensive molecular detection methods are essential for the early detection, diagnosis, and treatment of such disorders.

**Table 1:** Overview of clinical data of patients with CUL4B variants.

Ref	Cabezas et al. (2000)	North America	Tarpey et al. (2007)	Ravn et al. (2012)	Isidor et al. (2009)	Badura-Stronka et al. (2010)	Vulto-van Silfhout et al, (2015)	Londin et al. (2014)	Tzschach et al. (2015)	Weissbach et al. (2017)	Lopez et al. (2020)	Della Vecchia et al. (2023)	Magalhaes et al. (2023)	Europe	Nakamura et al. (2019)	Okamoto et al. (2017)	Zou et al. (2007)	This case*	Asia	Total
Area	North America		Europe	Europe	Europe	Europe	Europe	Europe	Europe	Europe	Europe	Europe	Europe		Asia	Asia	Asia	Asia		
Country	USA		UK	Denmark	France	Poland		Italy	Germany	Germany	Spain	Italy	Portugal		Japan	Japan	china	china		
Family	1	1	8	1	1	1	11	1	3	1	1	1	1		1	1	1	2		35
No. of affected	6	6	39	2	1	3	25	8	4	1	1	1	1		1	1	6	2		101
Abnormalities in cranial imaging	NR	/	0/22	NR	0/1	NR	10/15	8/8	NR	1/1	0/1	1/1	0/1		1/1	1/1	6/6	2/2		30/60
Neurological																				
Mental retardation	5/5	5/5	22/22	2/2	1/1	3/3	24/24	8/8	4/4	0/1	1/1	1/1	1/1	67/68	1/1	1/1	6/6	2/2	10/10	82/83
Speech delay	4/5	4/5	18/18	2/2	1/1	3/3	23/23	8/8	NR	0/1	1/1	1/1	1/1	58/59	1/1	1/1	6/6	2/2	10/10	72/74
Motor delay	5/5	5/5	5/5	2/2	1/1	NR	23/23	8/8	NR	0/1	1/1	1/1	1/1	42/43	1/1	1/1	6/6	2/2	10/10	57/58
Alogia	4/5	4/5	11/15	2/2	1/1	2/3	NR	8/8	NR	0/1	1/1	NR	1/1	26/32	0/1	1/1	5/6	0/1	6/9	36/46
Behavioral problems	3/5	3/5	12/15	1/2	1/1	3/3	13/22	NR	NR	1/1	1/1	1/1	NR	33/46	1/1	0/1	5/6	0/1	6/9	42/60
Tremor	4/6	4/6	11/13	2/2	1/1	2/3	9/20	NR	NR	0/1	0/1	1/1	1/1	27/43	0/1	1/1	1/5	1/2	3/9	34/57
Seizures	NR	/	8/11	2/2	0/1	NR	7/22	8/8	NR	NR	1/1	1/1	NR	27/46	1/1	0/1	4/5	1/2	6/9	33/55
Gait abnormality	4/5	4/5	6/12	2/2	1/1	NR	10/21	NR	NR	1/1	1/1	NR	NR	21/38	NR	0/1	6/6	0/1	6/8	31/51
Growth																				
Macrocephaly	0/5	0/5	8/11	2/2	0/1	1/3	7/22	NR	3/4	NR	1/1	1/1	NR	23/45	1/1	1/1	0/6	0/2	2/10	25/60
Short stature	5/5	5/5	7/11	2/2	1/1	2/3	17/22	8/8	2/4	0/1	1/1	0/1	1/1	41/55	1/1	1/1	6/6	2/2	10/10	56/70
Obesity	4/5	4/5	15/19	2/2	0/1	2/3	11/21	NR	3/4	0/1	0/1	0/1	NR	33/53	0/1	NR	NR	0/2	0/3	3,761
Craniofacial																				
High/prominent forehead	5/5	5/5	+	2/2	1/1	NR	13/19	NR	NR	NR	0/1	0/1	1/1	17/23	1/1	1/1	0/6	0/2	2/10	24/40
Malformed/abnormally positioned ears	0/5	0/5	+	2/2	1/1	3/3	17/19	8/8	1/4	NR	1/1	1/1	1/1	35/40	1/1	1/1	0/6	0/2	2/10	37/55
HSR/deep-set eyes/narrow palpebral fissures	+	/	+	2/2	0/1	3/3	17/22	8/8	NR	NR	1/1	1/1	0/1	32/39	0/1	1/1	0/6	0/2	1/10	33/49
Low nasal bridge/rounded tip	+	/	+	2/2	1/1	3/3	12/22	8/8	1/4	NR	1/1	1/1	0/1	29/43	1/1	1/1	0/6	1/2	2/10	32/53
Prominent lower lip/wide mouth	4/5	4/5	6/17	2/2	1/1	3/3	18/23	8/8	1/4	NR	1/1	0/1	1/1	41/61	0/1	1/1	0/6	0/2	1/10	46/76
Extremities																				
Brachydactyly/syndactyly 2nd-3rd toes	3/5	3/5	11/13	0/2	1/1	3/3	14/19	8/8	1/4	1/1	1/1	1/1	1/1	42/54	1/1	0/1	5/5	2/2	8/9	53/68
Small hands/small feet	5/5	5/5	NR	NR	NR	3/3	NR	8/8	NR	NR	NR	NR	1/1	12/12	NR	0/1	NR	0/2	0/3	17/20
Pes cavus	NR	/	7/8	2/2	1/1	NR	2/11	NR	NR	NR	0/1	1/1	NR	13/24	NR	NR	NR	0/1	0/1	13/25
Wasted lower-leg muscles	5/5	5/5	NR	NR	NR	NR	5/11	NR	NR	NR	1/1	0/1	NR	6/13	NR	NR	NR	0/2	0/2	11/20
Other																				
Hypogonadism/genital abnormalities	4/5	4/5	10/15	0/2	1/1	1/3	17/20	8/8	1/4	0/1	1/1	1/1	NR	40/56	NR	1/1	NR	0/2	1/3	45/64
Kyphosis/convex scoliosis	4/5	4/5	3/18	NR	1/1	1/3	6/18	NR	NR	1/1	1/1	1/1	1/1	15/44	NR	0/1	NR	0/2	0/3	19/52
Strabism	NR	/	NR	NR	1/1	NR	NR	NR	NR	NR	1/1	NR	NR	2/2	NR	NR	NR	0/2	0/2	2/4

NR, Not reported; HSR, hyperplastic supraorbital ridges. * The patients from China.

**Table 2:** Clinical data of presently and previously described patients with c.953_957delTTATA/p. Ile318fs*2.

Ref	Vulto-van Silfhout et al. (2015); Tarpey et al. (2007)	This study
MRI		
Cortical dysplasia	1/1	1/1
Enlarged fissure of Sylvius	1/1	0/1
Enlarged cavum veli interpositi	1/1	0/1
White matter lesions	0/1	0/1
Cavum septum pellucidum	0/1	0/1
CNS		
Stumbling	0/1	0/1
Abnormal muscle tone	0/1	1/1
Growth		
Birth weight (−2SD)	0/1	0/1
Birth height (−2SD)	0/1	0/1
Weight (−2SD)	/	1/1
Height (−2SD)	2/2	1/1
Head circumference (−2SD)	0/1	1/1
Microcephaly	/	0/1
Macrocephaly	0/1	0/1
Obesity	0/1	0/1
Neurological		
ID (level)	1/2 moderate; 1/2 unknown	1/1severe
Motor delay	2/2	1/1
Speech delay	2/2	1/1
Behavioral problems	2/2	NA
Attention deficit hyperactivity disorder	2/2	NA
Tremor	2/2	0/1
Seizures	0/2	0/1
Gait abnormality	2/2	0/1
Craniofacial dysmorphic features		
High/prominent forehead	0/2	0/1
Malformed/abnormally positioned ears	1/2	0/1
HSR/deep-set eyes/narrow palpebral fissures	2/2	0/1
Low nasal bridge/rounded tip	2/2	0/1
Prominent lower lip/wide mouth	2/2	0/1
Sagging cheeks	0/2	0/1
Extremities		
Brachydactyly/small hands/small feet	2/2	1/1
Syndactyly 2nd-3rd toes	/	0/1
Dysmorphic features of hands and feet		
Wasted lower leg muscles	0/1	0/1
Pes cavus	0/1	NA
Sandal notches	0/1	0/1
Others		
Hypogonadism/genital abnormalities	1/1	0/1
Gynecomastia	/	0/1
Cryptorchidism	0/1	0/1
Kyphosis	2/2	0/1
Convex scoliosis	0/2	0/1
Multiple lentigines	0/2	0/1
Strabism	2/2	0/1
Torticollis	2/2	0/1
Keratoconus	2/2	0/1

MRI, magnetic resonance imaging; CNS, central nervous system; HSR, hyperplastic supraorbital ridges; ID, intellectual disability.

In conclusion, this study identified two pathogenic variants in the *CUL4B* gene in two Chinese boys by using exome sequencing. Compared to previously reported cases, the two patients in our study exhibited severe developmental delay rather than other typical phenotypic features of Cabezas syndrome. We revealed that less pronounced clinical features were observed in patients with Cabezas syndrome in Chinese populations, especially in young children. It suggests that regular pediatric health check-ups and comprehensive molecular detection methods are essential for the early detection and accurate diagnosis of this type of disease.

## Data Availability

The datasets presented in this article are not readily available because of ethical and privacy restrictions. Requests to access the datasets should be directed to the corresponding authors.

## Footnotes

^1^ www.ncbi.nlm.nih.gov

^2^ www.hgmd.cf.ac.uk/ac/

^3^ liweilab.gene-qa.cn/index.html/

^4^ https://gnomad.broadinstitute.org/

^5^ https://www.internationalgenome.org/

^6^ https://www.ncbi.nlm.nih.gov/snp/?term=
